# Insights into the genetic diversity of *Mycobacterium tuberculosis* in Tanzania

**DOI:** 10.1371/journal.pone.0206334

**Published:** 2019-04-12

**Authors:** Liliana K. Rutaihwa, Mohamed Sasamalo, Aladino Jaleco, Jerry Hella, Ally Kingazi, Lujeko Kamwela, Amri Kingalu, Bryceson Malewo, Raymond Shirima, Anna Doetsch, Julia Feldmann, Miriam Reinhard, Sonia Borrell, Daniela Brites, Klaus Reither, Basra Doulla, Lukas Fenner, Sebastien Gagneux

**Affiliations:** 1 Swiss Tropical and Public Health Institute, Basel, Switzerland; 2 University of Basel, Basel, Switzerland; 3 Ifakara Health Institute, Bagamoyo, Tanzania; 4 Central Tuberculosis Reference Laboratory, Dar es Salaam, Tanzania; 5 National Tuberculosis and Leprosy Programme, Dar es Salaam, Tanzania; 6 Institute of Social and Preventive Medicine, University of Bern, Bern, Switzerland; University of Padova, Medical School, ITALY

## Abstract

**Background:**

Human tuberculosis (TB) is caused by seven phylogenetic lineages of the *Mycobacterium tuberculosis* complex (MTBC), Lineage 1–7. Recent advances in rapid genotyping of MTBC based on single nucleotide polymorphisms (SNP), allow for phylogenetically robust strain classification, paving the way for defining genotype-phenotype relationships in clinical settings. Such studies have revealed that, in addition to host and environmental factors, strain variation in the MTBC influences the outcome of TB infection and disease. In Tanzania, such molecular epidemiological studies of TB however are scarce in spite of a high TB burden.

**Methods and findings:**

Here we used SNP-typing to characterize a nationwide collection of 2,039 MTBC clinical isolates representative of 1.6% of all new and retreatment TB cases notified in Tanzania during 2012 and 2013. Four lineages, namely Lineage 1–4 were identified within the study population. The distribution and frequency of these lineages varied across regions but overall, Lineage 4 was the most frequent (n = 866, 42.5%), followed by Lineage 3 (n = 681, 33.4%) and 1 (n = 336, 16.5%), with Lineage 2 being the least frequent (n = 92, 4.5%). We found Lineage 2 to be independently associated with female sex (adjusted odds ratio [aOR] 2.14; 95% confidence interval [95% CI] 1.31 – 3.50, p = 0.002) and retreatment cases (aOR 1.67; 95% CI 0.95 – 2.84, p = 0. 065) in the study population. We found no associations between MTBC lineage and patient age or HIV status. Our sublineage typing based on spacer oligotyping on a subset of Lineage 1, 3 and 4 strains revealed the presence of mainly EAI, CAS and LAM families. Finally, we detected low levels of multidrug resistant isolates among a subset of 144 retreatment cases.

**Conclusions:**

This study provides novel insights into the MTBC lineages and the possible influence of pathogen–related factors on the TB epidemic in Tanzania.

## Introduction

Tuberculosis (TB) is the leading cause of mortality due to an infectious disease [1]. In 2017, an estimated 10 million people developed TB globally, with 1.6 million dying of the disease. Tanzania is among the thirty high burden countries, with a national average TB notification rate of 129 cases per 100,000; however, some regions show higher notification rates [2]. Like in most sub-Saharan African countries, the HIV epidemic contributes substantially to the high TB incidence in Tanzania, where a-third of the TB patients are co-infected with HIV [2]. Contrarily, drug-resistant TB is still low in this setting [3]. Other risk factors such as poverty also influence the epidemiology of TB in Tanzania [4].

Transmission of TB occurs via infectious aerosols, where upon exposure individuals can either clear the infection, develop active disease or remain latently infected [5]. The complex dynamics of TB infection and disease are determined by the environment, the host and the pathogen [6]. Seven main phylogenetic lineages of the *Mycobacterium tuberculosis* complex (MTBC) (Lineage 1–7) cause TB in humans [7]. These lineages are phylogeographically distributed, partially reflecting human migration histories [8–10]. Genomic differences among the MTBC strains translate into relevant biological and epidemiological phenotypes [11]. Epidemiologically speaking, these phenotypes are demonstrated by indicators such as transmission potential, disease severity and progression rates from infection to disease [12–15]. In general, strains of the widely distributed lineages, Lineage 2 and 4 or “generalists”, appear to be more virulent than those of the geographically restricted lineages, Lineage 5 and 6 or “specialists” [7,11].

Studying genotype-phenotype relationships requires understanding the genetic diversity of MTBC clinical strains in a given clinical setting. In Tanzania, few studies have described the genetic diversity of the MTBC [16–19]. These previous works used conventional genotyping tools such as the spacer oligonucleotide typing (spoligotyping) technique and revealed the presence of mainly the East African Indian (EAI), Central Asian (CAS) and Latin American Mediterranean (LAM) spoligo families, and the Beijing family reported only at the lowest frequencies. Based on phylogenetically robust techniques, which include single nucleotide polymorphisms (SNPs), the spoligo families correspond to Lineage 1, 3, 4 and 2, respectively. These previous studies from Tanzania are limited as they only focused on few specific geographical locations on the country and only one study profiled MTBC on a countrywide scale albeit with low sampling coverage [18]. Moreover, despite the invaluable contribution of techniques like spoligotyping in the molecular epidemiology field, such techniques are suboptimal for phylogenetically robust strain classification due to high rates of convergent evolution [20,21].

In this study, we applied for the first time a robust SNP typing method to classify the largest so far nationwide representative collection of clinical isolates to gain insights into unknown patterns of MTBC diversity in different regions of Tanzania. Given that only few studies have assessed and identified lineage-specific differences in clinical settings, we then looked for potential associations between the MTBC lineages and the available clinical and epidemiological characteristics of the patients in the study population.

## Material and methods

### Ethics statement

The study was approved by the National Tuberculosis and Leprosy Programme and the ethical clearance was provided by the National Institute for Medical Research of Tanzania (Dar es Salaam, Tanzania). The data in this study were analyzed anonymously.

### The National Tuberculosis and Leprosy Program routine drug surveillance system

Our study was based on a nationwide convenience sample of sputum smear positive new and retreatment TB cases diagnosed between 2012 and 2013 in Tanzania. The collection was obtained via a platform established for routine TB drug resistance surveillance by the National Tuberculosis Leprosy Program (NTLP) of Tanzania, covering health facilities in all geographical regions of the country. Briefly, smear positive sputa specimens from approximately 25% of new TB cases and from all retreatment cases were obtained for the drug resistance surveillance. To obtain 25% sputa from new cases each region was allocated four months per annum, where the respective health facilities in the region submitted sputa samples to zonal reference laboratories for culture. The zonal laboratories include the Central Tuberculosis Reference Laboratory (CTRL) in Dar es Salaam, Bugando Medical Center (BMC) in Mwanza and Kilimanjaro Christian Medical Center (KCMC) in Kilimanjaro, which serve the Coastal and Southern zone, the Lake zone, and the Northern zone, respectively. Isolates from the two zonal laboratories, BMC and KCMC were then sent to the CTRL for drug susceptibility testing (DST). For this study we included all the isolates we could retrieve from the culture archives at the CTRL.

### Study population and data collection

We included a total of 2,039 unique (single patient) culture-confirmed TB cases, each of whom we could retrieve the respective culture isolate from the CTRL culture archives. This study population represents 1.6% of all the estimated TB notified cases in the country between 2012 and 2013 (S1 Fig). We also obtained corresponding socio-demographic and clinical information collected during patients’ consultation at the respective health facilities. The demographic data collected included age, sex and geographical location of the patients, whereas clinical data included HIV status and disease category (i.e., new case and retreatment case).

### Processing of culture isolates

The smear positive sputa samples were cultured on Löwenstein Jensen (LJ) growth medium according to laboratory protocols. For this study, we included MTBC clinical isolates retrieved from archived LJ media. We then prepared heat inactivated samples for the retrieved clinical isolates by suspending MTBC colonies into 1ml sterile water and heat inactivate at 95°C for one hour.

### Molecular genotyping

We then classified the MTBC clinical isolates into main phylogenetic lineages by TaqMan real-time PCR according to standard protocols (Applied Biosystems, Carlsbad, USA) and as previously described [22]. Briefly, the TaqMan PCR uses fluorescently labeled allele-specific probes for singleplex SNP-typing that are specific for each MTBC lineage. For comparisons, we also performed 43-spacer spoligotyping on a membrane for a subset of representative MTBC clinical strains following standard protocols [23], since spoligotyping is still widely used as a gold standard for genotyping in similar settings. We randomly selected 107 samples out of the 2,039 representative of three lineages, Lineage 1, 3 and 4. We excluded Lineage 2 strains for this analysis given that such strains almost exclusively belong to the Beijing family. The clinical strains were assigned to spoligotype families using the online database SITVITWEB [24].

### Drug resistance genotyping

We selected a subset of 144 clinical isolates from the 321 retreatment cases to perform molecular drug resistance testing. We used a previously described multiplex polymerase chain reaction (PCR) to target the rifampicin resistance determining region of *rpoB* gene [25]. The PCR assay targets both the tuberculous and non-tuberculous *Mycobacteria* (MTBC and NTMs, respectively) *rpoB* gene, so we could also rule out the presence of non-tuberculous isolates in our study sample using the assay. The amplified *rpoB* gene product was confirmed by electrophoresis on a 2% agarose gel and sent for Sanger sequencing. We analyzed the resulting sequences by Staden software package [26] and using MTBC H37Rv *rpoB* gene as reference sequence.

### Statistical analysis

For statistical analysis, we applied descriptive statistics to delineate patients’ characteristics. We used Chi-square or Fisher’s exact tests for assessment of differences between groups in categorical variables, whenever applicable. We used univariate and multivariate logistic regression models to assess for the association between MTBC lineages and patients’ clinical and demographic characteristics. The associations were assessed for Lineage 2 compared to all other lineages (Lineages 1, 3 and 4), adjusting for age, sex, disease category and HIV status. All statistical analyses were performed in R 3.5.0 [27].

## Results

### Patients’ demographic and clinical characteristics

The patients’ demographic and clinical information in our study included age, sex, geographical location, HIV and disease category (new or retreatment case). Table 1 describes patients’ characteristics of the study population. The proportions of the observed and missing data for the study population are summarized in S2 Fig.

**Table 1:** Clinical and demographic characteristics of the TB cases.

**Characteristics**	**Valid Proportion %**	**Total (%)** **n = 2,039**
**Age, median (IQR)**		
35 (27–44)		
**Age groups (years)**		
Child age (< 15)	9.9	20 (1.0)
Young age (15–24)	29.7	312 (15.3)
Early adult (25–44)	48.0	1170 (57.4)
Late adult (45–64)	10.0	379 (18.6)
Old age (> 65)	2.5	73 (3.6)
Not available		85 (4.2)
	*total n = 1*,*954*	
**Sex**		
Female	32.4	645 (31.6)
Male	67.6	1346 (66.0)
Not available		48 (2.4)
	*total n = 1*,*991*	
**HIV status**		
Negative	67.7	1086 (53.3)
Positive	32.2	517 (25.4)
Indeterminate	0.06	1 (0.1)
Not available		435 (21.3)
	*total n = 1*,*604*	
**Patient category**		
New case	84.0	1,679 (82.3)
Retreatment	16.1	321 (15.7)
Not available		39 (1.9)
	*total n = 2*,*000*	
**Geographical zone**		
Central	1.1	22 (1.1)
Coastal	51.6	1,029 (50.5)
Lake	17.9	358 (17.6)
Northern	20.2	403 (19.8)
S. Highlands	8.1	162 (8.0)
Western	0.5	10 (0.5)
Zanzibar	0.6	12 (0.6)
Not available		43 (2.1)
	*total n = 1*,*996*	

IQR, interquartile range; valid proportion, proportion excluding missing values; Total n, all values including NA (not available).

Our study population consisted of TB patients ranging between the age of 2 and 89 years with a median age of 28 years (interquartile range [IQR] 27–44). To further probe the age distribution in the study population, we categorized the TB patients into five different age groups (Table 1). We detected approximately three-quarters of the TB cases to occur among the “young age” and “early adult” age groups. Further, our findings show that about 1.0% of the TB cases were pediatric cases (< 15 years).

Similar to other settings [1], we identified a higher proportion of male TB cases compared to female TB cases. However, the male-to-female ratio observed in our study population was higher than the national estimates for the two years of sampling (2.2:1 vs., 1.4:1). The striking gender imbalance among TB cases seems to peak at adolescence onwards and is less pronounced among pediatric TB cases (S1 Table). Additionally, a-third (32.2%, 517/1604) of the TB cases with available HIV status were HIV co-infected. In contrast, TB/HIV co-infected cases were more likely to be female (44.5%, CI 38.3–50.7% vs., 25.8%, 95% CI 20.6–31.0%) which is consistent with HIV being generally more prevalent in females than males in Tanzania [28]. We found that our study population comprised 16.1% (321/2000) of TB retreatment cases, which was four-fold higher than the overall countrywide notifications [29]. Finally, more than half (51.6%, 1029/1996) of the TB patients in our study population were diagnosed in the Coastal zone of Tanzania and about 40% were either diagnosed in the Lake and Northern zones. In addition to higher TB notification rates, the three former mentioned geographical zones contain the country’s zonal TB reference laboratories. The remaining 10% of the patients were diagnosed in any of the remaining four geographical zones of Tanzania.

### Main MTBC lineages in Tanzania

**Fig 1:**
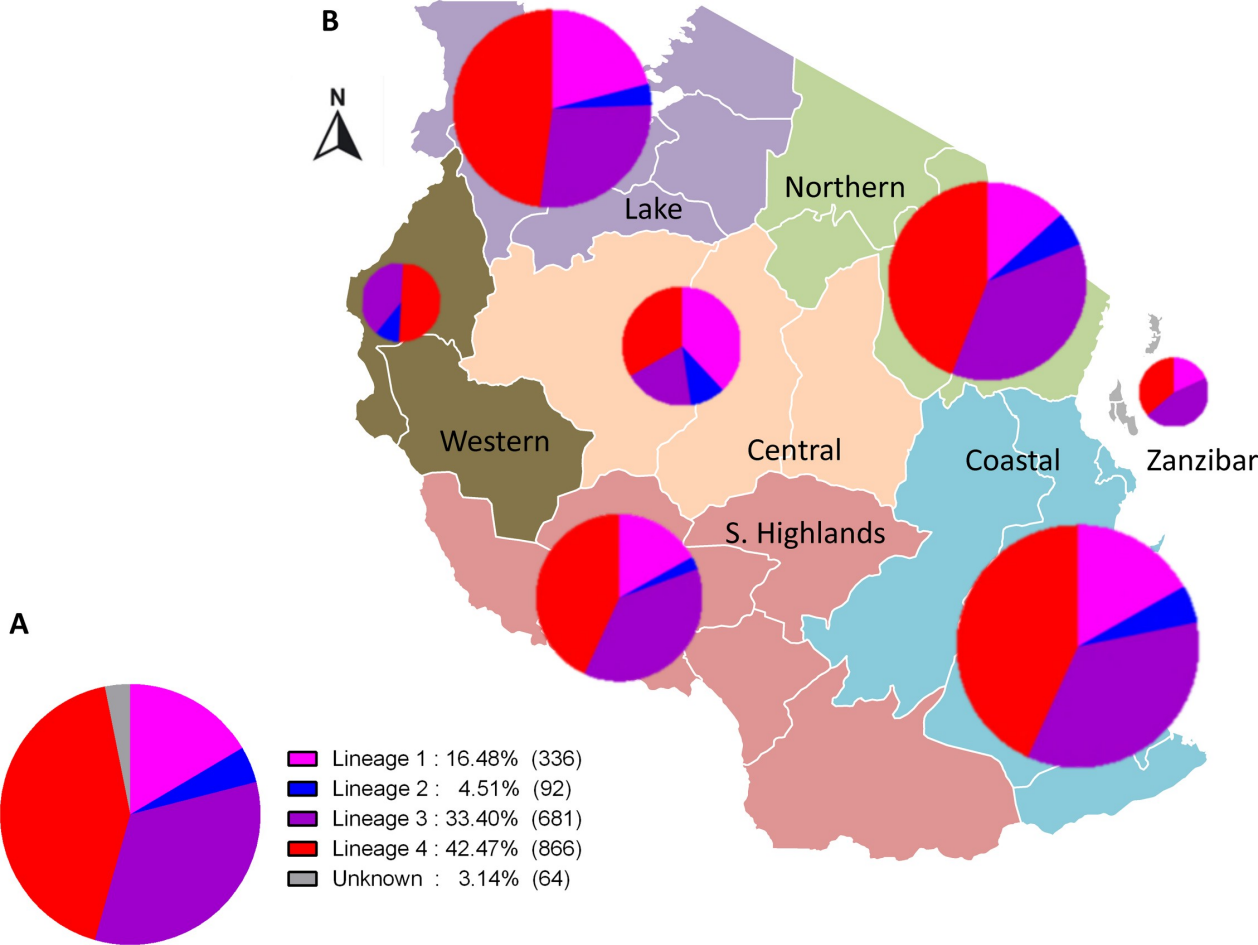
MTBC lineages in Tanzania. A. MTBC lineage classification of 2,039 nationwide clinical strains. B. MTBC lineage frequencies and geographical distribution in Tanzania.

Using SNP-typing, we detected four of the seven known MTBC lineages (Fig 1), albeit at varying proportions. In our study population, Lineage 4 and Lineage 3 were the most frequent (866, 42.5% and 681, 33.4%, respectively) followed by Lineage 1 (336, 16.5%). Lineage 2 was the least frequent (92, 4.5%). The remaining 64 clinical isolates (3.1%) could not be assigned to any of the MTBC lineages possibly because there was insufficient amount of DNA in the samples (below the detection limit). Of the seven geographical zones, four (Coastal, Northern, Lake and Southern Highlands) were highly represented with more than 100 clinical strains each (Table 2). The distribution of the MTBC lineages varied within the geographical zones (Fig 1 and S3 Fig). Our findings reveal that Lineage 1 strains were more frequent in the Lake zone compared to the overall average frequency (20.9% vs. 16.8%), whereas the frequency of Lineage 3 in this zone was lower (27.6% vs. 34.3%) compared to other geographical zones. By contrast, Lineage 4 was the most predominant in all geographical zones and showed relatively similar frequencies across the zones.

**Table 2:** MTBC lineage distribution across geographical regions in Tanzania.

Geographical Zone	Lineage				Total
	L1 (%)	L2 (%)	L3 (%)	L4 (%)	
Central	8 (38.1 )	2 (9.5 )	4 (19 )	7 (33.3 )	21
Coastal	168 (16.8 )	50 (5 )	350 (35 )	432 (43.2 )	1,000
Lake	72 (20.9 )	12 (3.5 )	95 (27.6 )	165 (48 )	344
Northern	52 (13.3 )	22 (5.6 )	145 (37 )	173 (44.1 )	392
S. Highlands	27 (16.9 )	4 (2.5 )	60 (37.5 )	69 (43.1 )	160
Western	0 (0 )	1 (10 )	4 (40 )	5 (50 )	10
Zanzibar	2 (18.2 )	0 (0 )	5 (45.5 )	4 (36.4 )	11
**Total**	329 (17 )	91 (4.7 )	663 (34.2 )	855 (44.1 )	1,938

L1, Lineage 1; L2, Lineage 2; L3, Lineage 3; L4, Lineage 4

### Sublineage classification

After we detected four main MTBC lineages, we next explored the respective subfamilies on a subset of Lineage 1, 3 and 4 strains using spoligotyping. Lineage 2 strains were excluded from this analysis since the global strains almost exclusively belong to one spoligotype family, Beijing with almost identical fingerprint pattern. We identified 24 spoligotypes (SITs; Spoligotype International Type) among the 107 clinical strains analyzed (S2 Table). Twenty six (24.3%) of the spoligo patterns had not been previously reported in the SITVITWEB database and therefore we assigned them as orphan spoligotypes. Several spoligotypes were identified within each of the three lineages. EAI5 was the common spoligotype among the Lineage 1 and CAS1_Kili spoligotype among the Lineage 3 strains. Within Lineage 4 strains, LAM, T, and H families were detected and the LAM subfamily, particularly LAM_ZWE was the most frequent.

### Association between lineage and patients’ characteristics

Having described the circulating main lineages of the MTBC we then assessed the relationship between these lineages and patients’ characteristics in the study population (Table 3). We detected a higher proportion of female sex among TB patients infected with Lineage 2 (52.1%) compared to those infected with the other three lineages (range from 31% to 34.5%, p = 0.009). Moreover, we observed that retreatment cases were frequently infected with Lineage 2 strains (26.8%), which was twofold higher compared to Lineage 1 and 4 strains (p < 0.001). We found no evidence for association between lineages and patients’ characteristics such as age and HIV status (Table 3).

**Table 3:** Frequency distribution of MTBC main lineages across patients’ characteristic groups.

Patient characteristics	Lineage			
	L1, n (%)	L2, n (%)	L3, n (%)	L4, n (%)
**Age group**				
Child age (< 15)	2 (0.7)	0 (0)	7 (1.3)	8 (1.2)
Young age (15–24)	44 (16.2)	10 (13.9)	79 (14.9)	112 (16.9)
Early adult (25–44)	156 (57.4)	44 (61.1)	336 (63.5)	378 (57.1)
Late adult (45–64)	57 (21.0)	15 (20.8)	85 (16.1)	140 (21.1)
Old age (> 65)	13 (4.8)	3 (4.2)	22 (4.2)	24 (3.6)
**Sex**				
Female	85 (31.3)	37 (51.4)	184 (34.8)	223 (33.7)
Male	187 (68.8)	35 (48.6)	345 (65.2)	439 (66.3)
**HIV status**				
Negative	184 (67.6)	45 (62.5)	349 (66.0)	459 (69.3)
Positive	88 (32.4)	27 (37.5)	180 (34.0)	203 (30.7)
**Patient category**				
New case	235 (86.4)	53 (76.6)	405 (76.6)	560 (84.6)
Retreatment	37 (13.6)	19 (26.4)	124 (23.4)	102 (15.4)
**Total**	272 (17.7)	72 (4.7)	529 (34.4)	662 (43.2)

L1, Lineage 1; L2, Lineage 2; L3, Lineage 3; L4, Lineage 4

Lineage 2 has previously been associated with retreatment cases, drug resistance and lately also with female sex [15,25]. We therefore investigated if similar associations exist in our study population using a subset of TB cases with complete clinical and demographic information (n = 1,535). To assess these associations we performed logistic regression analyses comparing Lineage 2 to all other lineages pooled together (Table 4). Our analyses revealed Lineage 2 to be independently associated with female sex (adjusted odds ratio [aOR] 2.14; 95% confidence interval [95% CI] 1.31 – 3.50, p = 0.002) and retreatment cases (aOR 1.67; 95% CI 0.95 – 2.84, p = 0.065). We did not detect any association between the lineages and patients’ age and the HIV status.

**Table 4:** Associations of patients’ clinical and demographic characteristics with MTBC Lineage 2 (n = 72) compared to all other lineages (n = 1,463).

Patient characteristics	Lineage 2	Unadjusted		Adjusted	
	n (%)	OR (95% CI)	p value	OR (95% CI)	p value
Age, median (IQR)	35 (28–44)			0.99 (0.97 – 1.01)	0.329
Female sex	37 (51.4)	2.09 (1.30–3.36)	0.002	2.14 (1.31 – 3.50)	0.002
Retreatment case	19 (26.4)	1.64 (0.93–2.76)	0.075	1.67 (0.95 – 2.84)	0.065
HIV positive	27 (37.5)	0.79 (0.49–1.31)	0.349	0.90 (0.55–1.51)	0.91
Observations		1,535		1,535	

IQR, Interquartile range; OR, Odds ratio; 95% CI, 95% confidence interval.

### Mutations within *rpoB* gene in retreatment cases

To investigate whether drug resistance was linked to a particular lineage, we included in total 144 out of 321 retreatment cases for drug resistance genotyping of the *rpoB* gene that confers resistance to rifampicin. Out of these, 112 (77.8%) had no mutations compared to the H37*Rv* reference gene and 15 (10.4%) contained at least one mutation, either synonymous (3/15) or non-synonymous (12/15) (S4 Fig and S3 Table). We could not determine mutation status in the *rpoB* gene of 17 (11.8%) retreatment cases due to PCR and sequencing failure. Among the 12 strains detected with non-synonymous *rpoB* mutations, five belonged to Lineage 2, four to Lineage 4, and three to Lineage 3 (S4 Table). Table 5 summarizes the non-synonymous *rpoB* mutations detected.

**Table 5:** Non-synonymous mutations detected on the *rpoB* gene among retreatment cases.

Lineage	*rpoB* mutation	Amino acid change	n	Source
**L2**	A1198G;C1349T	T400A;S450L	1	[30,31]
	C1333T	H445Y	1	[31]
	C1349T	S450L	3	[31]
**L3**	T1289C	L430P	1	[32]
	C1333T	H445Y	1	[31]
	C1349T	S450L	1	[31]
**L4**	A1334T	H445L	1	[31]
	G1333C	H445D	1	[31]
	C1294G;A1442G	Q432E;E481A	1	[32,33]
	C1333T	H445Y	1	[31]
**Total**			12	

## Discussion

In this study, we classified the countrywide collection of 2,039 MTBC isolates representing 1.6% of all smear positive new and retreatment TB cases notified during 2012 and 2013 in Tanzania. Our findings show that the MTBC strains among the study population are diverse, comprising four main phylogenetic lineages (Lineage 1–4) which occur throughout the country. Specifically, we found that Lineage 4 was the most frequent, followed by Lineage 3 and 1. Despite Lineage 2’s recent global dissemination [13], it was the least frequent in our study population. Finally, our analysis on the relationship between MTBC lineages and patients’ characteristics revealed associations of Lineage 2 with female sex and retreatment TB cases included in the study population.

Among the 7 human–adapted MTBC lineages, Lineage 4 is the most broadly distributed and occurs at high frequencies in Europe, the Americas and Africa [24,34]. In our study, we observe that TB epidemics in Tanzania are also predominated by Lineage 4, which is regarded as the most successful of MTBC lineages [34]. In general, the wide geographical range of Lineage 4 is postulated to be driven by a combination of its enhanced virulence, high rates of human migration linked to its spread and ultimately its ability to infect different human population backgrounds [34,35]. In contrast, Lineage 1 and 3 are known to be mainly confined to the rim of the Indian Ocean [7], which is consistent with our observation that nearly 50% of the MTBC strains included in the study belong to these two lineages. This high frequency of Lineage 1 and 3 likely reflects the long-term migrations between Eastern Africa and the Indian subcontinent [36]. In addition, the distribution and frequency of Lineage 1 and 3 in the mainland subset did not vary from that of the coastal region, suggesting spread via internal migrations. Lineage 1 was proposed to have evolved in East Africa prior disseminating out of the continent [10]. Based on this, one might expect higher frequencies of Lineage 1 in the region. Instead, the so called “modern” (TbD1−) lineages (4 and 3 in this case) could be dominating in Tanzania despite presumably being introduced into the African continent only after the first European contact [34,37]. This perhaps illustrates the ability of “modern” lineages to thrive in co-existence with the pre-existing “ancient” (TbD1+) lineages such as Lineage 1 in our case, perhaps because of the comparably higher virulence [14,38]. The neighboring countries of Tanzania on the other hand show comparable MTBC lineage composition to our study [39,40], suggesting common demographic histories and ongoing exchanges that resulted into distinct MTBC populations. Our findings would suggest the frequency of Lineage 2–Beijing in Tanzania, like in most parts of the continent except for South Africa [39,40] to be relatively low, despite the long-standing African-Asian contacts [40]. Evidence from recent studies show that Lineage 2–Beijing was only recently introduced into Africa [13,41].

The burden of TB disease is generally higher in males [1,42], rendering male sex as a potential risk factor for TB. Furthermore, the male bias among TB patients is also observed in settings with no obvious sex-based differences in health-seeking behavior [43]. Whilst we show similar trends in this study, our findings reveal that the proportion of females was higher among TB patients infected with Lineage 2. This finding is consistent with several other previous studies conducted in different settings [15,25,44]. Social and physiological factors predisposing males to higher risk of TB have been indicated [45]. On the one hand, these include risk behaviors such as substance abuse (alcoholism, tobacco smoking) and gender specific roles such as risk occupations (e.g., mining) that are male dominated and known to increase the risk for TB. On the other hand, genetic makeup and sex hormones might contribute to the differences in TB susceptibility among females and males, as epidemiological and experimental studies have suggested female sex hormones to be protective [45]. These observations would propose that the sex imbalance in TB emerges after the onset of puberty. Of note, we observe less sex imbalance in “child” age group (<15 years) which also corroborates the national notification rates [29]. However, this observation can be confounded by BCG vaccination which might be most effective in this age group. Despite the high prevalence of HIV among young females in sub-Saharan Africa [28] and HIV being the strongest risk factor for TB, TB burden remains higher in males. While social and physiological aspects play an important role, findings from this study and others previously conducted in Nepal and Vietnam [15,25] suggest that bacterial factors could disrupt the trends towards male bias in TB, a finding which warrants further investigation. Our hypothesis is that because of higher virulence, Lineage 2 strains are able to overcome the resistance poised by female sex which could explain the less pronounced sex imbalanced.

In addition to its association with female sex, we found that retreatment TB cases were more likely infected with Lineage 2. A retreatment case in our study population represented recurrent TB case either due to relapse or reinfection. We hypothesized that this observation was possibly linked to drug resistance, given the previous reported association between Lineage 2 and drug resistance [46]. However, we detected only 8.3% (12/144) of strains among the retreatment subset tested to contain mutations conferring resistance to rifampicin, five of which belonged to Lineage 2. These findings would suggest that retreatment cases included in this study are mainly driven by reinfection as opposed to treatment failure or relapse.

Finally, based on the age distribution of TB cases in our study, recent or ongoing transmission in high burden countries is implicated as the main contributor to the TB burden rather than disease reactivation (following longer latency periods) [47]. Additionally, an association with young age has been used as an epidemiological proxy for highly transmissible strains and faster rates of disease progression [48,49]. In this study, we did not detect any differences in median age of TB patients infected with different lineages (S5 Fig), an observation that could speak for high ongoing transmission rates in general, irrespective of lineage.

Our study is limited by focusing on a convenient collection of MTBC clinical isolates that could be retrieved from the culture archives, representing 1.6% of all TB cases notified in 2012 and 2013. Given that our findings are based on a limited number of TB cases, the results particularly those related to associations between lineages and patients’ characteristics should be taken with caution as the strength or lack of such associations could likely be affected by the sampling. In addition, most of the geographical zones were underrepresented which could in turn underestimate the respective regional lineage composition and the overall countrywide distribution. Unfortunately, data on drug susceptibility based on other methods such as Xpert MTB/RIF, phenotypic DST and Line Probe Assay (LPA) were unavailable, which could have complemented the drug resistance genotyping performed on a limited subset of the retreatment cases. Systematic sampling would allow for better resolution on the distribution patterns, the frequencies and on epidemiological features of MTBC lineages, which might partially determine the regional specific epidemics.

In conclusion, this study addresses for the first time the countrywide MTBC population structure based on robust SNP-typing. We show that MTBC population in Tanzania is diverse with four of the seven known lineages detected. This study sets the stage for further in depth investigations on epidemiological impact of MTBC lineages in Tanzania.

## Supporting information

S1 Fig Flowchart illustrating estimated notified TB cases in 2012 and 2013 (dashed lines) and the study population (solid line). (PDF)

S2 Fig Patients’ data included in the study. Proportion of observed and missing data for the variables included in the study.
(TIF)

S3 Fig MTBC lineage proportions. Distribution of MTBC lineages across different regions of Tanzania. Size of the circle is proportional to the number of isolates analyzed from the regions.
(TIF)

S4 Fig Flowchart of genotyped strains for *rpoB* mutations. A subset of MTBC strains from retreatment cases included for *rpoB* drug resistance genotyping.
(PDF)

S5 Fig Patients’ age distribution across MTBC lineages. The age distributions of TB patients grouped by infecting MTBC lineage.
(TIFF)

S1 Table Sex distribution across different age groups of TB patients. (PDF)

S2 Table Spoligotype patterns of a subset of MTBC clinical strains. (PDF)

S3 Table Mutations detected in the *rpoB* gene. (PDF)

S4 Table Distribution of *rpoB* mutations across the four MTBC lineages. (PDF)

## References

[1] WHO. Global tuberculosis report Geneva: World Health Organization 2017.

[2] NTLP. Annual report for 2016. Dar es Salaam. 2016;1–48.

[3] Nagu TJ, Aboud S, Mwiru R, Matee M, Fawzi W, Mugusi F. Multi Drug and Other Forms of Drug Resistant Tuberculosis Are Uncommon among Treatment Naïve Tuberculosis Patients in Tanzania. Surolia A, editor. PLoS One. 2015;10(4):e0118601 10.1371/journal.pone.0118601 25849784 PMC4388561

[4] MoHSW. First tuberculosis prevalence survey in the United Republic of Tanzania. 2013;

[5] Rieder HL. Epidemiologic Basis of Tuberculosis Control First edition 1999. 1999.

[6] Comas I, Gagneux S. The Past and Future of Tuberculosis Research. Manchester M, editor. PLoS Pathog. 2009;5(10):e1000600 10.1371/journal.ppat.1000600 19855821 PMC2745564

[7] Gagneux S. Ecology and evolution of Mycobacterium tuberculosis. Nat Rev Microbiol. 2018;16(4):202–13. 10.1038/nrmicro.2018.8 29456241

[8] Gagneux S, DeRiemer K, Van T, Kato-Maeda M, de Jong BC, Narayanan S, et al Variable host-pathogen compatibility in Mycobacterium tuberculosis. Proc Natl Acad Sci. 2006;103(8):2869–73. 10.1073/pnas.0511240103 16477032 PMC1413851

[9] Fenner L, Egger M, Bodmer T, Furrer H, Ballif M, Battegay M, et al HIV Infection Disrupts the Sympatric Host–Pathogen Relationship in Human Tuberculosis. Gibson G, editor. PLoS Genet. 2013;9(3):e1003318 10.1371/journal.pgen.1003318 23505379 PMC3591267

[10] Comas I, Coscolla M, Luo T, Borrell S, Holt KE, Kato-Maeda M, et al Out-of-Africa migration and Neolithic coexpansion of Mycobacterium tuberculosis with modern humans. Nat Genet. 2013;45(10):1176–82. 10.1038/ng.2744 23995134 PMC3800747

[11] Coscolla M. Biological and Epidemiological Consequences of MTBC Diversity In: Strain variation in the Mycobacterium tuberculosis complex:Its role in biology, epidemiology and control. Springer, Cham; 2017 p. 95–116. 10.1007/978-3-319-64371-7_5 29116631

[12] Hanekom M, van der Spuy GD, Streicher E, Ndabambi SL, McEvoy CRE, Kidd M, et al A Recently Evolved Sublineage of the Mycobacterium tuberculosis Beijing Strain Family Is Associated with an Increased Ability to Spread and Cause Disease. J Clin Microbiol. 2007;45(5):1483–90. 10.1128/JCM.02191-06 17360841 PMC1865897

[13] Cowley D, Govender D, February B, Wolfe M, Steyn L, Evans J, et al Recent and Rapid Emergence of W‐Beijing Strains of Mycobacterium tuberculosis in Cape Town, South Africa. Clin Infect Dis. 2008;47(10):1252–9. 10.1086/592575 18834315

[14] Stavrum R, PrayGod G, Range N, Faurholt-Jepsen D, Jeremiah K, Faurholt-Jepsen M, et al Increased level of acute phase reactants in patients infected with modern Mycobacterium tuberculosis genotypes in Mwanza, Tanzania. BMC Infect Dis. 2014;14(1):309. 24903071 10.1186/1471-2334-14-309 PMC4057905

[15] Holt KE, McAdam P, Thai PVK, Thuong NTT, Ha DTM, Lan NN, et al Frequent transmission of the Mycobacterium tuberculosis Beijing lineage and positive selection for the EsxW Beijing variant in Vietnam. Nat Genet. 2018;50(6):849–56. 10.1038/s41588-018-0117-9 29785015 PMC6143168

[16] Eldholm V, Matee M, Mfinanga SGM, Heun M, Dahle UR. A first insight into the genetic diversity of Mycobacterium tuberculosis in Dar es Salaam, Tanzania, assessed by spoligotyping. BMC Microbiol. 2006;6(1):76. 16970826 10.1186/1471-2180-6-76 PMC1592105

[17] Kibiki GS, Mulder B, Dolmans WM, de Beer JL, Boeree M, Sam N, et al M. tuberculosis genotypic diversity and drug susceptibility pattern in HIV- infected and non-HIV-infected patients in northern Tanzania. BMC Microbiol. 2007;7(1):51. 17540031 10.1186/1471-2180-7-51 PMC1913919

[18] Mfinanga SGM, Warren RM, Kazwala R, Kahwa A, Kazimoto T, Kimaro G, et al Genetic profile of Mycobacterium tuberculosis and treatment outcomes in human pulmonary tuberculosis in Tanzania. Tanzan J Health Res. 2014;16(2):58–69. 26875299 10.4314/thrb.v16i2.1

[19] Mbugi E V., Katale BZ, Siame KK, Keyyu JD, Kendall SL, Dockrell HM, et al Genetic diversity of Mycobacterium tuberculosis isolated from tuberculosis patients in the Serengeti ecosystem in Tanzania. Tuberculosis. 2015;95(2):170–8. 10.1016/j.tube.2014.11.006 25522841 PMC4364622

[20] Comas I, Homolka S, Niemann S, Gagneux S. Genotyping of Genetically Monomorphic Bacteria: DNA Sequencing in Mycobacterium tuberculosis Highlights the Limitations of Current Methodologies. Litvintseva AP, editor. PLoS One. 2009;4(11):e7815 10.1371/journal.pone.0007815 19915672 PMC2772813

[21] Fenner L, Malla B, Ninet B, Dubuis O, Stucki D, Borrell S, et al “Pseudo-Beijing”: Evidence for Convergent Evolution in the Direct Repeat Region of Mycobacterium tuberculosis. Sechi LA, editor. PLoS One. 2011;6(9):e24737 10.1371/journal.pone.0024737 21935448 PMC3172296

[22] Stucki D, Malla B, Hostettler S, Huna T, Feldmann J, Yeboah-Manu D, et al Two New Rapid SNP-Typing Methods for Classifying Mycobacterium tuberculosis Complex into the Main Phylogenetic Lineages. Mokrousov I, editor. PLoS One. 2012;7(7):e41253 10.1371/journal.pone.0041253 22911768 PMC3401130

[23] Kamerbeek J, Schouls L, Kolk A, van Agterveld M, van Soolingen D, Kuijper S, et al Simultaneous detection and strain differentiation of Mycobacterium tuberculosis for diagnosis and epidemiology. J Clin Microbiol. 1997;35(4):907–14. 9157152 10.1128/jcm.35.4.907-914.1997 PMC229700

[24] Demay C, Liens B, Burguière T, Hill V, Couvin D, Millet J, et al SITVITWEB–A publicly available international multimarker database for studying Mycobacterium tuberculosis genetic diversity and molecular epidemiology. Infect Genet Evol. 2012;12(4):755–66. 10.1016/j.meegid.2012.02.004 22365971

[25] Malla B, Stucki D, Borrell S, Feldmann J, Maharjan B, Shrestha B, et al First Insights into the Phylogenetic Diversity of Mycobacterium tuberculosis in Nepal. Sola C, editor. PLoS One. 2012;7(12):e52297 10.1371/journal.pone.0052297 23300635 PMC3530561

[26] Staden R. The Staden sequence analysis package. Mol Biotechnol. 1996;5(3):233–41. 8837029 10.1007/BF02900361

[27] R Core Team. R: A Language and Environment for Statistical Computing. R Foundation for Statistical Computing, Vienna; 2018.

[28] Hegdahl HK, Fylkesnes KM, Sandøy IF. Sex Differences in HIV Prevalence Persist over Time: Evidence from 18 Countries in Sub-Saharan Africa. Faragher EB, editor. PLoS One. 2016;11(2):e0148502 10.1371/journal.pone.0148502 26841112 PMC4739589

[29] National Tuberculosis and Leprosy Programme (NTLP). Annual report for 2013. Dar es Salaam. 2013.

[30] Phelan J, Coll F, McNerney R, Ascher DB, Pires DE V, Furnham N, et al Mycobacterium tuberculosis whole genome sequencing and protein structure modelling provides insights into anti-tuberculosis drug resistance. BMC Med. 2016;14(1):31. 27005572 10.1186/s12916-016-0575-9 PMC4804620

[31] Walker TM, Kohl TA, Omar S V, Hedge J, Del C, Elias O, et al Whole-genome sequencing for prediction of Mycobacterium tuberculosis drug susceptibility and resistance: a retrospective cohort study. Lancet Infect Dis. 2015;15:1193–202. 10.1016/S1473-3099(15)00062-6 26116186 PMC4579482

[32] Miotto P, Cabibbe AM, Borroni E, Degano M, Cirillo DM. Role of Disputed Mutations in the rpoB Gene in Interpretation of Automated Liquid MGIT Culture Results for Rifampin Susceptibility Testing of Mycobacterium tuberculosis. J Clin Microbiol. 2018;56(5):e01599–17. 10.1128/JCM.01599-17 29540456 PMC5925711

[33] Heyckendorf J, Andres S, Köser CU, Olaru ID, Schön T, Sturegård E, et al What Is Resistance? Impact of Phenotypic versus Molecular Drug Resistance Testing on Therapy for Multi- and Extensively Drug-Resistant Tuberculosis. Antimicrob Agents Chemother. 2018;62(2):1550–67. 10.1128/AAC.01550-17 PMC5786814 29133554

[34] Stucki D, Brites D, Jeljeli L, Coscolla M, Liu Q, Trauner A, et al Mycobacterium tuberculosis lineage 4 comprises globally distributed and geographically restricted sublineages. Nat Genet. 2016;48(12):1535–43. 10.1038/ng.3704 27798628 PMC5238942

[35] Coscolla M, Gagneux S. Consequences of genomic diversity in Mycobacterium tuberculosis. Semin Immunol. 2014;26(6):431–44. 10.1016/j.smim.2014.09.012 25453224 PMC4314449

[36] O’Neill MB, Shockey AC, Zarley A, Aylward W, Eldholm V, Kitchen A, et al Lineage specific histories of Mycobacterium tuberculosis dispersal in Africa and Eurasia. bioRxiv. 2018;210161. 10.1111/mec.15120 PMC6660993 31066139

[37] Comas I, Hailu E, Kiros T, Bekele S, Mekonnen W, Gumi B, et al Population Genomics of Mycobacterium tuberculosis in Ethiopia Contradicts the Virgin Soil Hypothesis for Human Tuberculosis in Sub-Saharan Africa. Curr Biol. 2015;25(24):3260–6. 10.1016/j.cub.2015.10.061 26687624 PMC4691238

[38] Portevin D, Gagneux S, Comas I, Young D. Human Macrophage Responses to Clinical Isolates from the Mycobacterium tuberculosis Complex Discriminate between Ancient and Modern Lineages. Bessen DE, editor. PLoS Pathog. 2011;7(3):e1001307 10.1371/journal.ppat.1001307 21408618 PMC3048359

[39] Mbugi E V., Katale BZ, Streicher EM, Keyyu JD, Kendall SL, Dockrell HM, et al Mapping of Mycobacterium tuberculosis Complex Genetic Diversity Profiles in Tanzania and Other African Countries. Sreevatsan S, editor. PLoS One. 2016;11(5):e0154571 10.1371/journal.pone.0154571 27149626 PMC4858144

[40] Chihota VN, Niehaus A, Streicher EM, Wang X, Sampson SL, Mason P, et al Geospatial distribution of Mycobacterium tuberculosis genotypes in Africa. Arez AP, editor. PLoS One. 2018;13(8):e0200632 10.1371/journal.pone.0200632 30067763 PMC6070189

[41] Rutaihwa LK, Menardo F, Stucki D, Gygli SM, Ley SD, Malla B, et al Multiple introductions of the Mycobacterium tuberculosis Lineage 2 Beijing into Africa over centuries. bioRxiv. 2018;413039.

[42] Guerra-Silveira F, Abad-Franch F. Sex Bias in Infectious Disease Epidemiology: Patterns and Processes. Nishiura H, editor. PLoS One. 2013;8(4):e62390 10.1371/journal.pone.0062390 23638062 PMC3634762

[43] Rhines AS. The role of sex differences in the prevalence and transmission of tuberculosis. Tuberculosis. 2013;93(1):104–7. 10.1016/j.tube.2012.10.012 23219235

[44] Buu TN, Huyen MN, Lan NTN, Quy HT, Hen N V, Zignol M, et al The Beijing genotype is associated with young age and multidrug-resistant tuberculosis in rural Vietnam. Int J Tuberc Lung Dis. 2009;13(7):900–6. 19555542

[45] Nhamoyebonde S, Leslie A. Biological Differences Between the Sexes and Susceptibility to Tuberculosis. J Infect Dis. 2014;209(suppl 3):S100–6. 24966189 10.1093/infdis/jiu147

[46] Borrell S, Gagneux S. Infectiousness, reproductive fitness and evolution of drug-resistant Mycobacterium tuberculosis. Int J Tuberc Lung Dis. 2009;13(12):1456–66. 19919762

[47] Yates TA, Khan PY, Knight GM, Taylor JG, McHugh TD, Lipman M, et al The transmission of Mycobacterium tuberculosis in high burden settings. Lancet Infect Dis. 2016;16(2):227–38. 10.1016/S1473-3099(15)00499-5 26867464

[48] de Jong BC, Hill PC, Aiken A, Awine T, Antonio M, Adetifa IM, et al Progression to Active Tuberculosis, but Not Transmission, Varies by Mycobacterium tuberculosis Lineage in The Gambia. J Infect Dis. 2008;198(7):1037–43. 10.1086/591504 18702608 PMC2597014

[49] Borgdorff MW, van Soolingen D. The re-emergence of tuberculosis: what have we learnt from molecular epidemiology? Clin Microbiol Infect. 2013;19(10):889–901. 10.1111/1469-0691.12253 23731470

