# Supplementary figures and images for: Insights into the genetic diversity of Mycobacterium tuberculosis in Tanzania
Source: PLoS One. 2019 Apr 12;14(4):e0206334. doi: 10.1371/journal.pone.0206334 (PMC6461268; doi:10.1371/journal.pone.0206334)

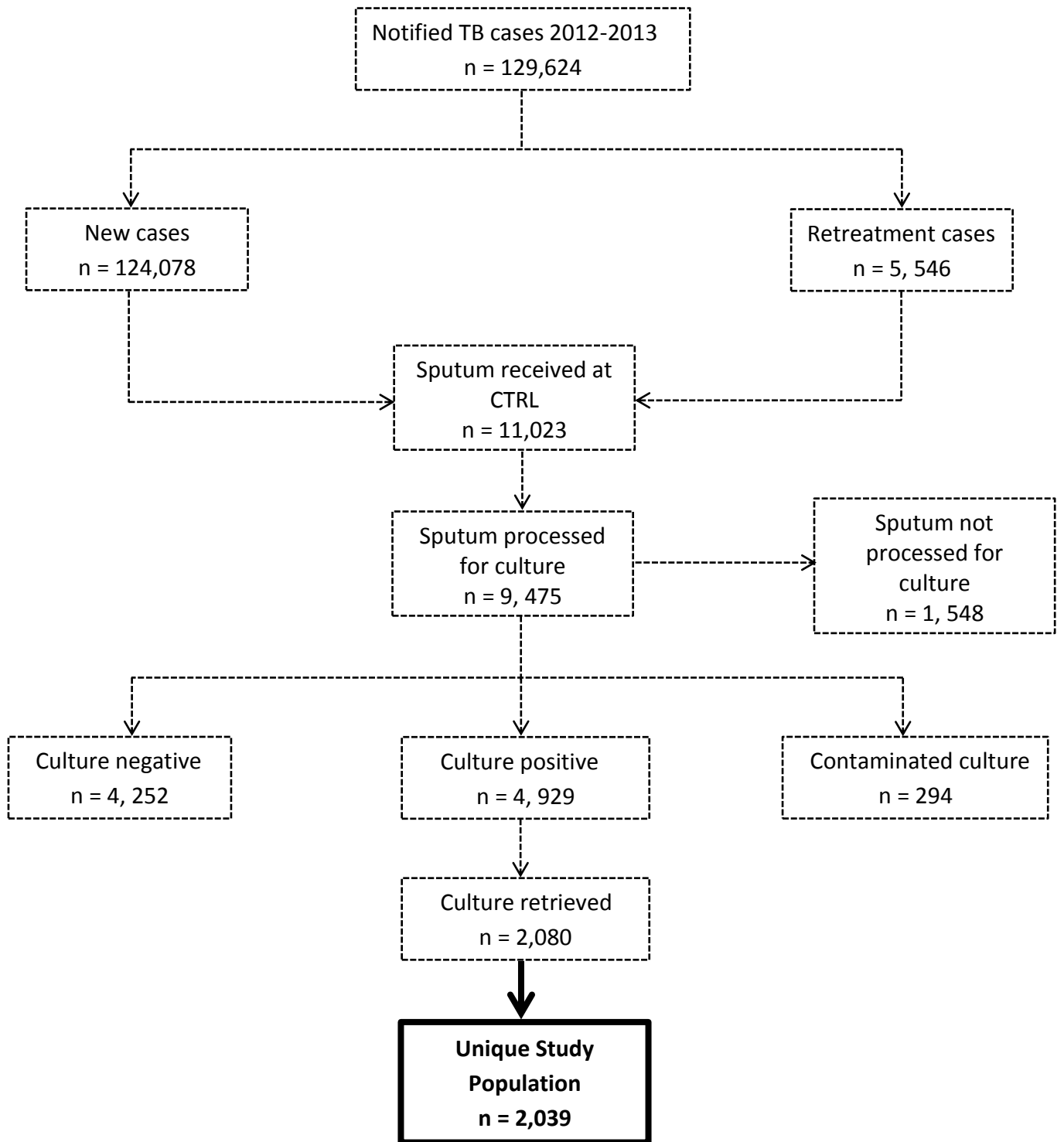

Supplement: S1 Fig — (PDF) [file pone.0206334.s001.pdf]

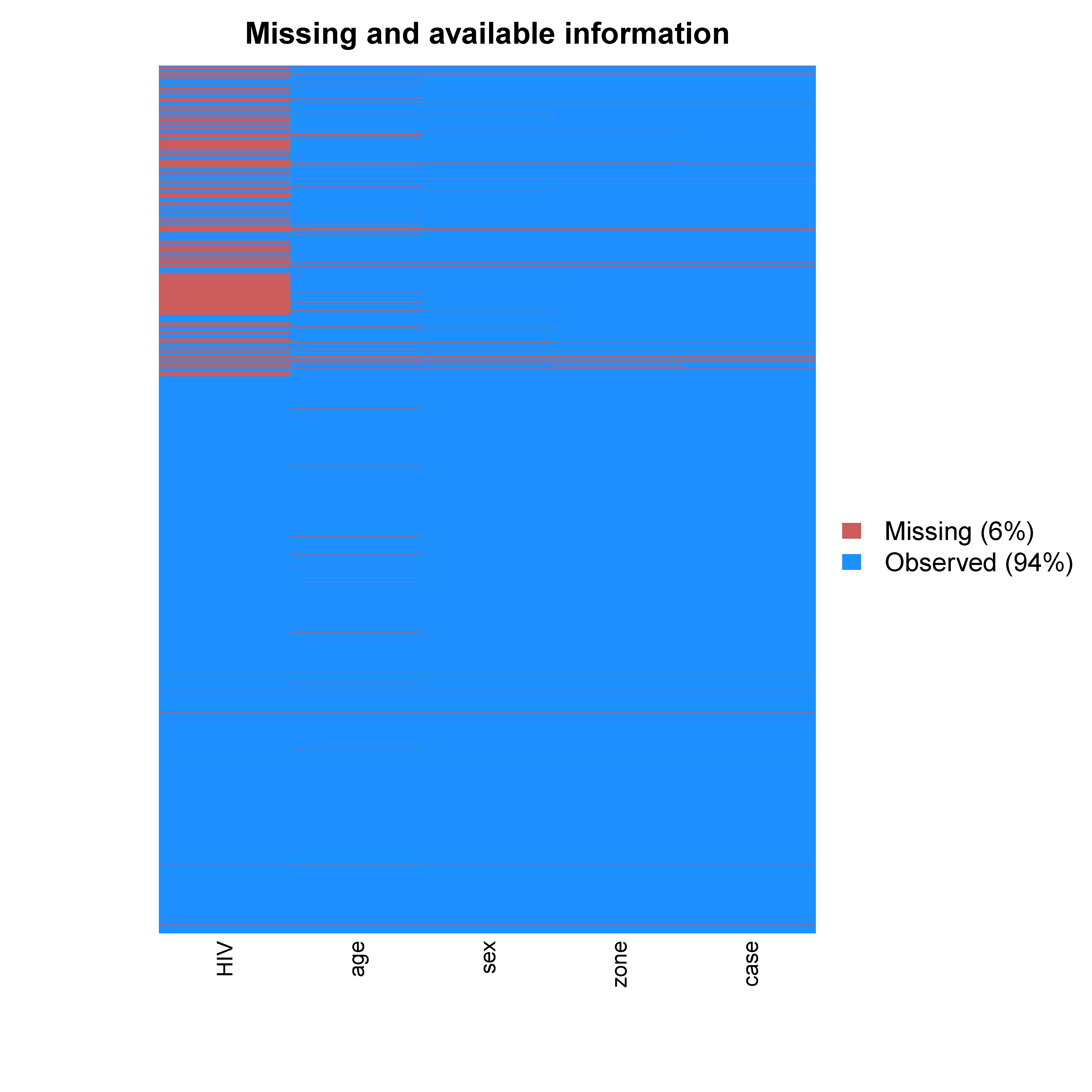

Supplement: S2 Fig — Proportion of observed and missing data for the variables included in the study. (TIF) [file pone.0206334.s002.tif]

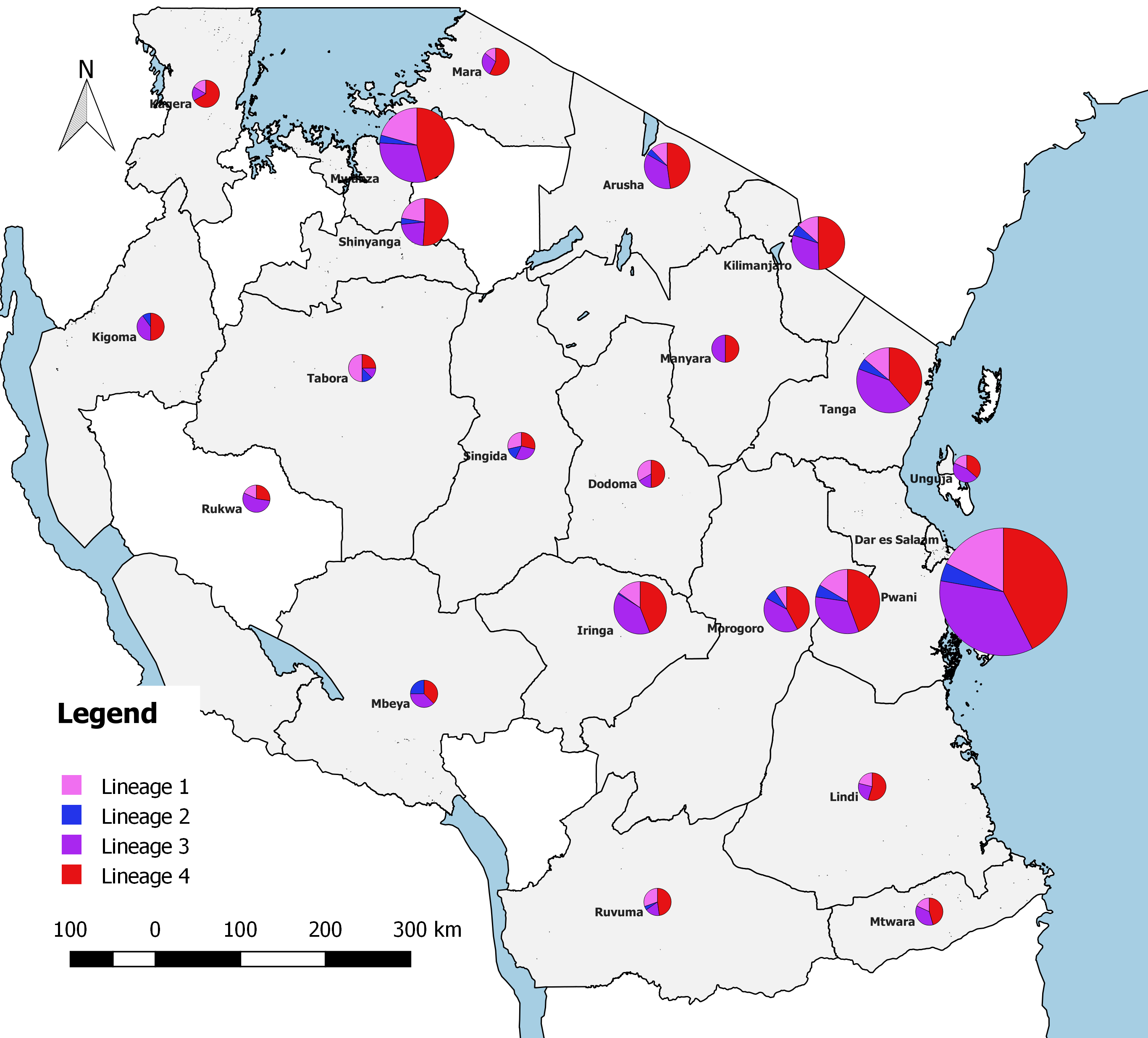

Supplement: S3 Fig — Distribution of MTBC lineages across different regions of Tanzania. Size of the circle is proportional to the number of isolates analyzed from the regions. (TIF) [file pone.0206334.s003.tif]

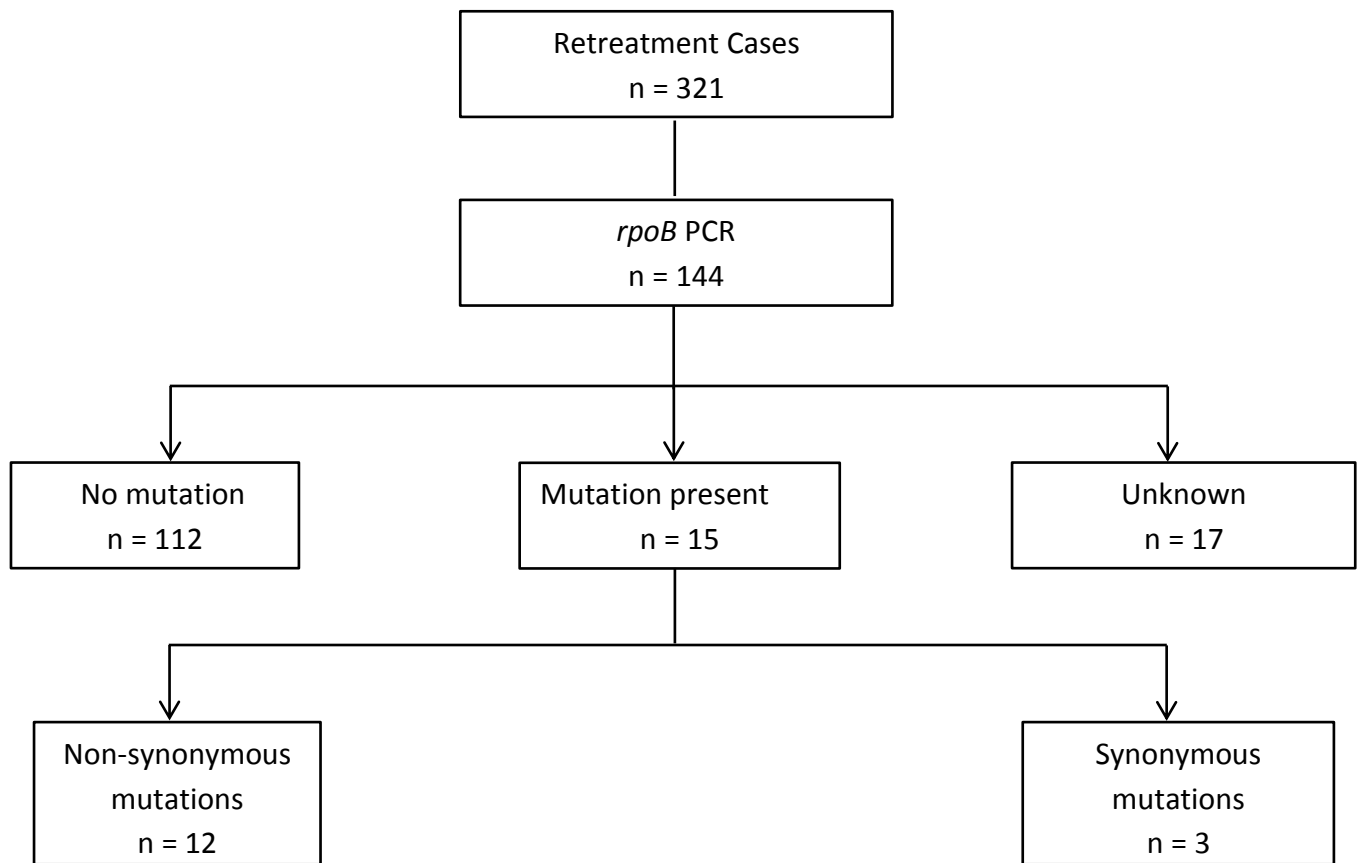

Supplement: S4 Fig — A subset of MTBC strains from retreatment cases included for rpoB drug resistance genotyping. (PDF) [file pone.0206334.s004.pdf]

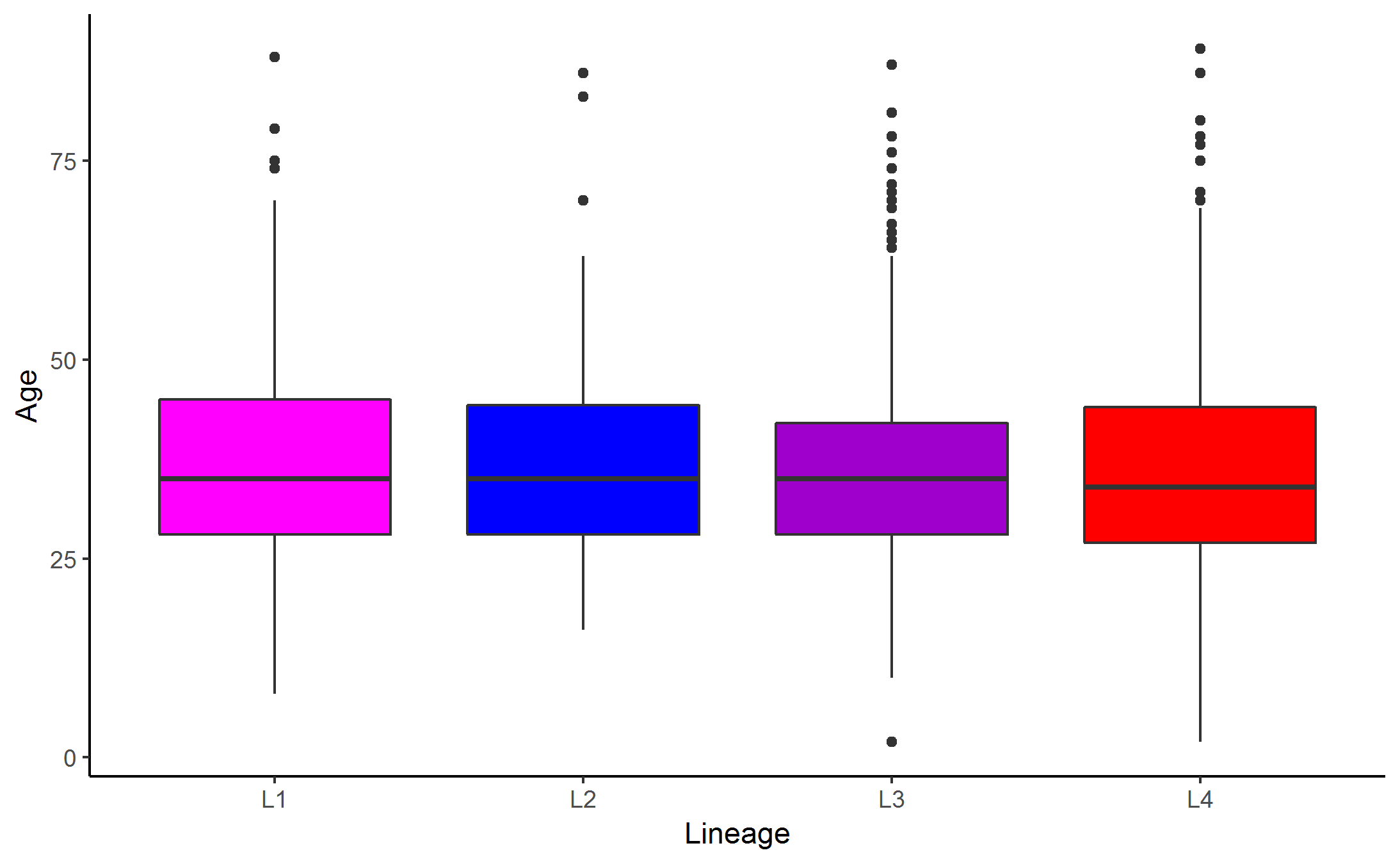

Supplement: S5 Fig — The age distributions of TB patients grouped by infecting MTBC lineage. (TIFF) [file pone.0206334.s005.tiff]
