# Supplementary material for: Insights into the genetic diversity of Mycobacterium tuberculosis in Tanzania
Source: PLoS One. 2019 Apr 12;14(4):e0206334. doi: 10.1371/journal.pone.0206334 (PMC6461268; doi:10.1371/journal.pone.0206334)
Supplement: S1 Table — (PDF) [file pone.0206334.s006.pdf]

Table S1. Sex distribution across the age groups.

Age group	Sex		**Total**
	F	M	
Child age (< 15)	82 (42.5)	111 (57.5)	193
Young age (15 - 24)	208 (35.9)	372 (64.1)	580
Early adult (25 - 44)	290 (30.9)	647 (69.1)	937
Late adult (45 – 64)	46 (23.5)	150 (76.5)	196
Old age (> 65)	13 (27.1)	35 (72.9)	48
Total	639 (32.7)	1,315 (67.3)	1,954
