# Supplementary material for: Insights into the genetic diversity of Mycobacterium tuberculosis in Tanzania
Source: PLoS One. 2019 Apr 12;14(4):e0206334. doi: 10.1371/journal.pone.0206334 (PMC6461268; doi:10.1371/journal.pone.0206334)
Supplement: S2 Table — (PDF) [file pone.0206334.s007.pdf]

Table S2. Spoligotype patterns for selected *M. tuberculosis* clinical isolates.

Lineage	n	Spoligotype pattern (spacer 1-43)	SIT	Spoligoytpe name
1	2	■■■■■■■■■■■□□□□□□□□□□□□□■■■■□□□□■□■■□■■■■■■	924	EAI5
1	10	■□□□□□□□□□□□□■■■■■■■■■■■■■■■□□□□■□■■■■■■■■■	8	EAI5
1	7	■□□■■■■■■■■■■■■■■■■■■■■■■■■■□□□□■□■■■■■■■■■	126	EAI5
1	1	□□□■■■■■■■■■■■□■■■■■■■■■■■■■□□□□■□■■■■■□■■■	1734	EAI1_SOM
1	1	■■■■■■■■■■■■■■■■■■■■■■■■■■■■□□□□■□■■■■■□■■■	48	EAI1_SOM
1	1	■□□□□□□□□□□□□■■■■■■■■■■■■■■■□□□□■□■■■■■■■□■	2593	Unknown
1	1	■□□□□□□□□□□□□■■■■■■■■■■■■□■■□□□□■□■■■■■■■■■	Orphan	Unknown
1	1	■■■□□□□□□□■■■■■■■■■■■■□□■■■■□□□□■□■■■■■■■■■	Orphan	Unknown
1	1	■□□■■■■■■■■■■■■■■■■■■■■■■■■■□□□□■□■■■■■□□□□	Orphan	Unknown
1	1	■□□■■■■■■■■□□□□■■■■■■■■■■■■■□□□□■□■■■■■■■■■	Orphan	Unknown
1	1	■■■■■■■■■■■■■■□■■■■■■■■□□■■□□□□■■□□■■■■■■■■■	Orphan	Unknown
1	1	□□□□□□□□□□□□□■■■■■■■■■■■■■■■□□□□■□■■■■■■■■■	Orphan	Unknown
1	1	■□□■■■■■■■■■■■■■■■■■■■■■■■■■□□□□■□■■■□■■■■■	Orphan	Unknown
1	1	■■■■■■■■■■■□■■■■■■■■■■□■■■■■□□□□■□■■□■■■■■■	Orphan	Unknown
1	1	■□□■■■■■■■■■■■■■■■■■■■■■■■■■□□□□■□■■■■■■□■■	Orphan	Unknown
1	1	■□□■■■■■■■■■■■■■■■■■■■■■■■■■□□□□■□■■□■■■■■■	Orphan	Unknown
1	1	■□□■■■■■■■■■□■■■■■■■■■■■■■■■□□□□■□■■□□□■■■■	Orphan	Unknown
3	28	■■■□□□□■■□■■■■■■■■■□□□□□□□□□□□□□□□□■■■■■■■■	21	CAS1_Kili
3	1	■■■□□□□■■□■■□■■■■■■□□□□□□□□□□□□□□□□■■■■■■■■	1765	CAS1_Kili
3	1	■■■□□□□■■□■■■■■■■■■■■■□□□□□□□□□□□□■■■■■■■■■	1942	CAS1-Delhi
3	1	■■■□□□□■■□■■■■□■■■■■■■□□□□□□□□□□□□■■■■■■■■■	Orphan	Unknown
3	1	■■□□□□□□□□□□□□□■■■■■□□□□□□□□□□□□□□■■□□□□■■□	Orphan	Unknown
3	1	■■■■■■□■■■■■■■■■■■■■□□□□□□□□□□■■□□□■■■■■■■■	Orphan	Unknown
4	10	■■■■■■■■■■■■■■■■■■■■□□□□■■□□□□■■□□□□■■■■■■■	59	LAM11_ZWE
4	1	■■■■■■■■■■■■■■■■□■■■□□□□■■□□□□■■□□□□■■■□■■■	2196	LAM11_ZWE
4	1	■■■■■■■■■■■■■■□■■■■■□□□□■■□□□□■■□□□□■■■■■■■	2268	LAM11_ZWE
4	1	■■■■■■■■■■■□■■■■■■■■□□□□■■□□□□■■□□□□■■■■■■■	2492	LAM11_ZWE
4	1	■■■■■■■■■■■■□■■■■■■■□□□□■■□□□□■■□□□□■■■■■■■	1471	LAM11_ZWE
4	1	■■□□□□□□■■■■■■■■■■■■□□□□■■□□□□■■□□□□■■■■■■■	1607	LAM11_ZWE
4	1	■■■■■■■■■■■■■■■■■■■■□□□□■■□□□□■■□□□□■■■□■■■	815	LAM11_ZWE
4	2	■■■■■■■■■■■■■■■■■■■■□□□□■■■■■■■■□□□□■■■■■■■	42	LAM9
4	1	■■■■■■□□■■■■■■■■■■■■□□□□■■■■■■■■□□□□■■■■■■■	1064	LAM9
4	3	■■■■■■■■■■■■■■■■■■■■■■■■■■■■■■■■□□□□■■■■■■■	53	T1
4	1	■■□■■■■■■■■■■■■■■■■■■■■■■■■■■■■■□□□□■■■■■■■	196	T1
4	1	■■■■■■■■■■■■□□□□■■■■■■■■■■■■■■■■□□□□■■■■■■■	102	T1
4	2	■■■■■■■■■■■■□■■■■■■■■■■■■■■■■■■■□□□□■■■□■■■	73	T2 & T3
4	1	■■■■■■■■■■■■■■■■■■■■■■■■■□□□□□□■□□□□■■■□■■■	62	H1
4	1	■■■■■■■■■■■■■■■■■■■■■■■■□□□□□□□□□□□□□□□□□□□	46	U (likely H)
4	1	■■□□■■■■■□■■■■■■□■■■■■■■■■■■■■■■□□□□■■■□■■□	Orphan	Unknown
4	1	■■□□■■■■■□■■■■■■■■■■■■■■■■■■■■■■□□□□■■■□■■□	Orphan	Unknown
4	1	■■■■■■■□■■■■■□■■■■■■□□□□■■□□□□■■□□□□■■■□■■■	Orphan	Unknown
4	1	■■■□□■□□■■□□■□□■■■■■□□□□□■□□□□■■□□□□■■□■■■■	Orphan	Unknown
4	1	■■□□■■■■■□■■■■■■■■■■■■■■■■■■■■■■□□□□■■■□■■□	Orphan	Unknown

4	1	■■■■■■■■■■□□□□□□■□□■■■■■■■■■■■■■□□□□■■■■■■■	Orphan	Unknown
4	1	■■□□■■■■■□■■■■■■■■■■■■■■■■■■■■■■□□□□■■■□■■□	Orphan	Unknown
4	1	■■■□□□□□□□□□□□□■■■□□□□□□□□□□□□□□□□□□□□□□□□□	Orphan	Unknown
4	1	■■□□■■■■■□■■■■■■■■■■■■■■■■■■■■■■□□□□■■■□■■□	Orphan	Unknown
4	1	■■■□■■■■■□■■□■■■■■■■□□□□■■□□□□■■□□□□■■■■■■■	Orphan	Unknown
4	1	■■■■■■■■■■□□□□■■■■■■■■■■■■■■■■■■□□□□■■■■■■■	Orphan	Unknown
4	1	■■□□■■■■■□■■■■■■■■■■■■■■■■■■■■■□□□□□■■■□■■□	Orphan	Unknown
