# Supplementary material for: Insights into the genetic diversity of Mycobacterium tuberculosis in Tanzania
Source: PLoS One. 2019 Apr 12;14(4):e0206334. doi: 10.1371/journal.pone.0206334 (PMC6461268; doi:10.1371/journal.pone.0206334)
Supplement: S4 Table — (PDF) [file pone.0206334.s009.pdf]

Table S3. Mutations detected on the *rpoB* gene

patient	Lineage	rpoB mutation	Amino acid change
315	L2	A1198G;C1349T	T400A;S450L
446	L3	C1386T	No change
470	L4	G1683A	No change
620	L4	A1334T	H445L
626	L4	G1333C	H445D
718	L2	C1333T	H445Y
719	L4	G1683A	No change
720	L2	C1349T	S450L
1649	L3	T1289C	L430P
1816	L2	C1349T	S450L
1822	L3	C1333T	H445Y
1834	L2	C1349T	S450L
1843	L3	C1349T	S450L
1927	L4	C1294G;A1442G;G1683A	Q432E;E481A; No change
1949	L4	C1333T	H445Y
